# Laryngeal Pressure Estimation With a Recurrent Neural Network

**DOI:** 10.1109/JTEHM.2018.2886021

**Published:** 2018-12-27

**Authors:** Pablo Gómez, Anne Schützenberger, Marion Semmler, Michael Döllinger

**Affiliations:** Division of Phoniatrics and Pediatric AudiologyDepartment of Otorhinolaryngology, Head and Neck SurgeryUniversity Hospital Erlangen, Friedrich-Alexander University Erlangen-Nürnberg91054ErlangenGermany

**Keywords:** High-speed video, inverse problem, recurrent neural networks, vocal fold dynamics, voice physiology

## Abstract

Quantifying the physical parameters of voice production is essential for understanding the process of phonation and can aid in voice research and diagnosis. As an alternative to invasive measurements, they can be estimated by formulating an inverse problem using a numerical forward model. However, high-fidelity numerical models are often computationally too expensive for this. This paper presents a novel approach to train a long short-term memory network to estimate the subglottal pressure in the larynx at massively reduced computational cost using solely synthetic training data. We train the network on synthetic data from a numerical two-mass model and validate it on experimental data from 288 high-speed *ex vivo* video recordings of porcine vocal folds from a previous study. The training requires significantly fewer model evaluations compared with the previous optimization approach. On the test set, we maintain a comparable performance of 21.2% versus previous 17.7% mean absolute percentage error in estimating the subglottal pressure. The evaluation of one sample requires a vanishingly small amount of computation time. The presented approach is able to maintain estimation accuracy of the subglottal pressure at significantly reduced computational cost. The methodology is likely transferable to estimate other parameters and training with other numerical models. This improvement should allow the adoption of more sophisticated, high-fidelity numerical models of the larynx. The vast speedup is a critical step to enable a future clinical application and knowledge of parameters such as the subglottal pressure will aid in diagnosis and treatment selection.

## Graphical abstract

In the clinical routine the air pressure in the larynx is usually inaccessible. We trained a neural network on a numerical model to estimate the pressure from endoscopic videos at minimal computational cost.

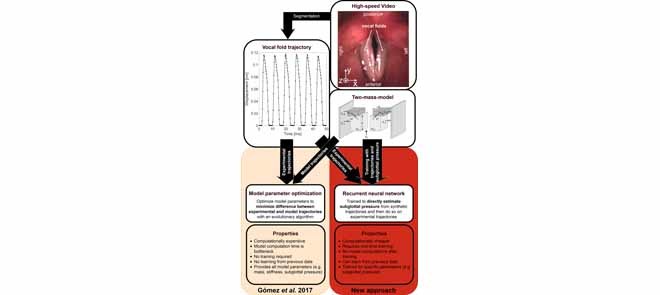

## I. Introduction

Disorders of the human voice have an underappreciated prevalence and impact on the affected and society in general. Bhattacharyya [1], e.g., found that about 7.6% of the US population reported voice problems in a time-frame of 12 months and those affected reported more than twice the amount of lost workdays compared to those not reporting voice problems.

One problem facing voice research is that the understanding of the process of phonation, i.e. the process of the vocal folds producing sound through oscillation, is hindered by its physical complexity and the confining human anatomy.

We propose a way to train a long short-term memory (LSTM) [2] network to solve the inverse problem of estimating the laryngeal or so called subglottal pressure, i.e. the air pressure below the vocal folds, for given high-speed video recordings of the vocal fold dynamics. The subglottal pressure is a highly important factor in the phonatory process. It causes the driving force of the vocal fold oscillation and can be indicative of a voice disorder [3]–[6]. There have been approaches for the measurement of the subglottal pressure using tools such as a so-called Rothenberg mask [7], which estimates subglottal pressure from the airflow or by performing a tracheal puncture [8], but easier access would aid the clinical routine [9].

We demonstrated in a previous study [10] that it is possible to estimate the subglottal pressure in a numerical inverse problem. However, the previous results indicate that the optimization would benefit from a more sophisticated model, which is not computationally feasible. Alternative approaches that lessen the computational burden are required. To circumvent the costly optimization and simulation runs, i.e. model evaluations, we trained a LSTM using solely synthetically generated training data from a numerical model to predict the subglottal pressure and then tested it on the same experimental data from the previous study. Having the necessary amount of labeled training data to enable a deep learning approach is often a problem [11], which was tackled by using synthetic data for the training. Overall, this work features the following contributions:

• An experimentally validated approach for subglottal pressure estimation using neural networks instead of a classical optimization
• Compared to a classical optimization approach comparable accuracy at vastly reduced computational cost
• First approach with potential for real-time estimation of the subglottal pressure from vocal fold trajectories
• First approach that can reasonably enable utilization of sophisticated models (e.g. 2D Navier-Stokes models) for parameter estimation by training with them
• Showcasing possibility to train neural networks for problems in voice research with numerical models to make up for very limited data

## II. Related Work

Inverse problems to estimate the vocal fold parameters have been a topic of voice research for the last fifteen years and numerous studies employed these approaches [12]–[15]. Only limited research has been conducted on the applicability of deep learning approaches to these problems, although recently there has been great interest to apply deep learning approaches in medicine. The suggested approach builds on work from both areas, research on vocal fold parameter estimation and recent deep learning approaches.

### A. Background

The human voice features oscillation of the vocal folds with up to 350 Hz during normal phonation. This necessitates sophisticated measurement techniques such as videostrobos-copy [16], [17] or high-speed video endoscopy [18]–[20]. Measurements are further impaired by the limiting anatomy of the human larynx. Therefore, complex experimental setups and numerical models are necessary to further the understanding of the human phonation.

Recent research pursues several avenues ranging from sophisticated experimental research [21], [22] to numerical simulation [23], [24]. One aim of the research is gaining insight on the physical properties such as mass or stiffness of the vocal folds as they are critical for understanding the physical process [15], [25]–[27]. Knowledge of those properties is paramount, especially as parameters such as the subglottal pressure have been linked to dysphonia, the abnormal or impaired voice [3]–[6].

### B. Modeling and Inverse Problems in Voice Research

Modeling plays an essential role in understanding the human phonatory process by providing insight and information. It is also used to estimate vocal fold parameters through an inverse problem. This is achieved through the optimization of the parameters of a numerical model to minimize the difference between model behavior and a recording of real vocal fold dynamics. If the numerical model is physically sound and the optimization successful, it is possible to infer the parameters of the real vocal folds. First suggested by Döllinger *et al.* [12] this approach has been successfully used to, e.g., estimate the subglottal pressure [10] or study disorders such as unilateral vocal fold paralysis [14].

The most commonly used numerical model in these endeavors is the two-mass model by Ishizaka and Flanagan [28]. Higher fidelity models based, e.g., on a Lattice Boltzmann approach [29] or Navier-Stokes airflow model [30]–[32] are available, but usually computationally too expensive to employ in an inverse problem. Detailed reviews of available models are given by Erath *et al.* [33] and Alipour *et al.* [23].

### C. Deep and Transfer Learning in Inverse Problems

Recent years have seen great advances in deep learning, many of those in the domain of computer vision. In this field it has become common to use pre-trained convolutional neural networks (CNNs). This reduces the computational burden caused by the training and allows employing the CNNs on problem sets of limited size. Many previous studies investigated the generalization abilities and transferability of features in CNNs [34]–[36]. The ability of neural networks to transfer to similar problem domains has been researched intensively for other network architectures and in applied situations as well [11], [37]. CNNs have also been employed in image-related inverse problems such as denoising or image reconstruction [38], [39]. McCann *et al.* [38] also describe the difficulties arising from the limited amount of real training data available in a biomedical context and ways to generate data. However, as shown, e.g., by Jaderberg *et al.* [40] it is possible to train with synthetically created data, if insufficient real data are available and thereby utilize the generalization potential of neural networks.

Finally, the application domains of deep learning are still being explored and recent results serve to highlight the potential of employing neural networks as surrogate models in numerical simulations. Ling *et al.* [41], e.g., demonstrated a successful application approximating a high-fidelity model in fluid mechanics and Paganini *et al.* [42] approximated simulation results in particle physics.

## III. Methods and Procedures

While the precise procedure used in the inverse problem formulation of previous studies varies [10], [12], [15], [43], the general idea remains similar. Given some noise-affected data of real vocal fold oscillations $y$, we want to find a configuration $q$ for a numerical model $G$ such that

\begin{equation*} y \approx G(q).\tag{1}\end{equation*}

If the problem is solved successfully, i.e. the difference between $y$ and $G(q)$ is minimized and $G$ is capable of capturing the physical reality, then the physical parameters of $G$ will, ideally, resemble the real physical parameters. It should be noted that this is in fact an ill-posed inverse problem, where uniqueness and existence of the found solution are not guaranteed.

This approach was employed in a previous study [10] to estimate the subglottal pressure present in *ex vivo* experiments using porcine larynges. In this study, we are relying on the same numerical model used to generate labeled training data and instead train a neural network to estimate the subglottal pressure. Training on synthetic data is necessary due to the limited amount of available experimental data. Several steps are taken to enable this transfer of training on synthetic data followed by testing on experimental data.

### A. The Two-Mass Model

**FIGURE 1.**
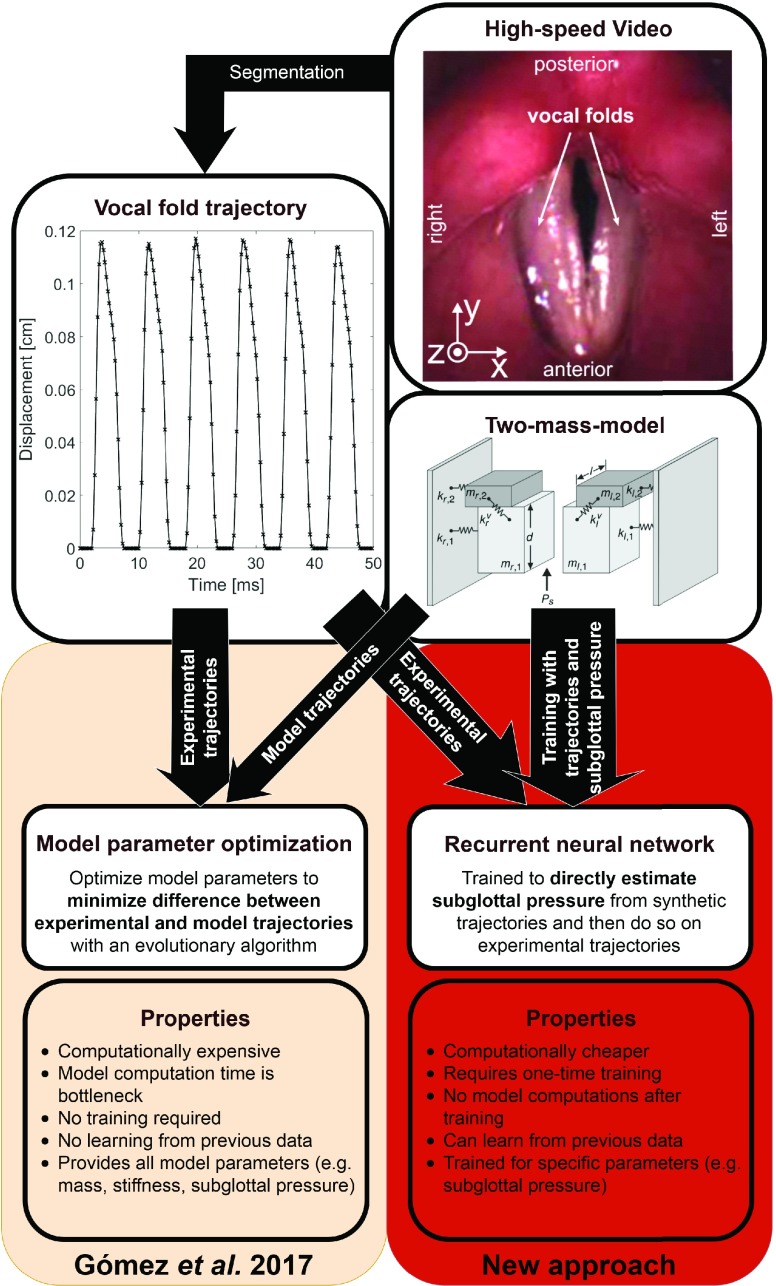
Depiction of the two-mass model (Original image by Schwarz *et al.* [14]), HSV data, an experimental trajectory and the previous and new inversion approach.

The employed model [10] is a slight modification of the original two-mass model (2MM) by Ishizaka and Flanagan [28], which was later simplified by Steinecke and Herzel [44]. It is a lumped-mass model that found extensive use in voice research [12], [13], [15], [45]. Figure 1 contains a depiction of the 2MM. As in the previous study [10], the movement of the mass $(s,i)$ with $s$ and $i$ describing the side (left, right) and plane (lower, upper) is governed by the equation

\begin{equation*} m_{s,i} \ddot {x}_{s,i} = F_{s,i}^{a} + F_{s,i}^{v} + F^{c}_{s,i} + F_{s,i}^{d} \tag{2}\end{equation*}

with the forces

\begin{align*}&F_{s,i}^{a} \, \text {- non-linear anchor spring force}\\&F_{s,i}^{v} \, \text {- vertical coupling force}\\&c_{s,i} \, \text {- non-linear force due to collision} \\&F_{s,i}^{d} \, \text {- non-linear driving force}.\end{align*}

The 2MM is very popular due to its extensive ability to replicate and produce realistic vocal fold dynamics while being computationally cheap. However, the model has certain limitations such as only describing medio-lateral oscillation and reliance on a relatively simplistic Bernoulli airflow model [46], [47]. A more sophisticated model relying on, e.g., a Navier-Stokes airflow model is necessary to capture effects such as the source-filter interaction of the supgraglottal tract and vocal fold dynamics [48], [49]. Furthermore, it does not account for the often present posterior gap, which has been shown to be associated with the subglottal pressure [50], [51]. A detailed discussion of conceptual limitations is given by Erath *et al.* [33]. The presented approach is not reliant on a particular model, but the 2MM was used to obtain results that can be directly compared to the previous study [10], which employed it as well. The authors are unaware of another experimentally validated baseline to compare to and the 2MM has seen widespread application to estimate vocal fold parameters [10], [12], [13], [15], [24].

As in the work of Döllinger *et al.* [12] the output of the 2MM are the trajectories $T_{l}[n]$ and $T_{r}[n]$, which describe the lateral, i.e. in $x$-direction, position of the masses at time step $n$. Figure 1 contains an example of a vocal fold trajectory.

### B. Experimental Data and Estimation Baseline

The experimental data used in this work were originally obtained by Birk *et al.* [22], where the experimental setup used to collect the data is described in detail. They recorded the oscillation of six *ex vivo* porcine larynges during different experimental setups. A previous study investigated an optimization of the parameters of a 2MM and we used the same recordings as in that study [10]. This dataset was chosen for two reasons. First, it contains trajectories and corresponding subglottal pressure values. Secondly, the previous study provided a baseline accuracy for subglottal pressure estimation with an optimization of model parameters that can be compared to. The final dataset contains 288 trajectory pairs, i.e. lateral displacement of the respective vocal fold, and corresponding subglottal pressure. Each trajectory contains $N=400$ data points sampled at $F_{s}= 4000$ Hz, i.e. 100 ms of continuous and regular oscillation. Observed frequencies of the oscillation were between 70 and 281 Hz.

Note, that the recording and the process of extracting the trajectories from the recordings introduce noise. Birk *et al.* [22] provide a detailed description of the noise affecting the experimental procedure and the previous study [10] discusses other factors introducing noise. *Ex vivo* data from animals such as sheep, dogs or pigs is regularly analyzed in voice research as the restricting anatomy of the human larynx inhibits *in vivo* measurement of parameters such as the subglottal pressure [22], [23], [52], [53].

### C. The Parameter Estimation

The actual parameter estimation relies on minimizing the difference between the 2MM trajectories $T_{l}$ and $T_{r}$ and experimental trajectories $T_{l}^{exp}$ and $T_{r}^{exp}$. Experimental trajectories can be obtained from the segmentation of high-speed video recordings of the vocal fold oscillations as described by Döllinger *et al.* [54]. The difference between the trajectories is described by an objective function $\Gamma $. Various formulations of $\Gamma $ have been suggested and this work relies on a previously introduced one [55]. It describes a phase-invariant mean absolute percentage error (MAPE) between the experimental and 2MM trajectories.

For each pair of experimental trajectories $T_{l}^{exp}$ and $T_{r}^{exp}$ an optimization on the $d$-dimensional parameter space $Q = [Q_{i}^{l},Q_{i}^{u}]^{d}$, where $Q_{i}^{l}$ is the lower bound of the $i$-th 2MM parameter with $0 < i \leq d$ and $Q_{i}^{u}$ is the upper bound respectively, is performed. After the optimization converged or is otherwise deemed successful, the estimation of the 2MM parameters is given by

\begin{equation*} \hat {q} = \underset {q \in Q}{\mathrm {argmin}} \quad \Gamma \,.\tag{3}\end{equation*}

Suitable parameters were studied in a previous study [55]. Typical parameters affect model masses, spring stiffnesses, subglottal pressure, collisions strength or spring damping factors [10], [15]. Previous studies with a 2MM used between $d=3$ [12] and $d=14$ parameters [55].

The optimization is highly dependent on the properties of $\Gamma $, which is usually a non-convex and non-smooth function. It is therefore common to employ evolutionary algorithms or other derivative-free optimization methods to minimize it [10], [15]. In preceding works [10], [55] we found that using the objective function $\Gamma $ at least 10 000 2MM evaluations are necessary for convergence, going up to over 100 000 2MM evaluations for a higher number of parameters $d$. This critically limits the computational complexity of the employed numerical model as for each recording these model evaluations have to be performed anew. However, the accuracy of the parameter estimation is also limited by the simplicity of the model. Consequently, a way to employ a more complex model with less evaluations is needed to improve accuracy and allow future clinical applicability.

### D. Estimation With a Deep Learning Approach

An alternative approach to solve the inverse problem is presented here. As discussed in Section II applications of deep learning approaches to problems in medicine are often hindered by the limited amount of training data available and one of the solutions to this has been the application of transfer learning or domain adaptation approaches [11], [56]. In particular, this work relies on training a LSTM to estimate the subglottal pressure present in a recording from the trajectories $T_{l}$ and $T_{r}$. Each trajectory is a one-dimensional time domain signal. From that signal, the target in this work was to estimate a scalar value that for synthetic, i.e. 2MM trajectories describes the subglottal pressure $P_{s}$ in the 2MM and in case of the experiment the mean subglottal pressure measured in the experiment $P_{exp}$. Alternative tools that, e.g., provide confidence intervals such as Kalman filtering are applicable to this problem too [57], but the LSTM was chosen as it requires no prior assumptions and has seen widespread application to sequential and especially speech data [58].

This work focused on the subglottal pressure to compare the performance of the LSTM on the dataset used in a previous study [10], where the subglottal pressure for 288 high-speed *ex vivo* video recordings of oscillations of porcine vocal folds was estimated. Note that only the dataset of the previous study was used as, to the authors’ knowledge, no other dataset providing a baseline comparison was available.

The previous approach achieved a mean absolute error (MAE) of 284 Pa or 173 Pa, if one calibrates the numerical model accounting for an underestimation of the subglottal pressure, which was found in the study. The corresponding MAPE was 27.5% or 17.7% respectively. To achieve these results, a total of $4.32 \cdot 10^{7}$ model evaluations were computed.

The idea behind our work is that if we can train a LSTM using training data that is synthetically generated with the 2MM, it is conceivable that we might achieve a similar accuracy estimating the subglottal pressure as by employing the 2MM in the optimization procedure described previously [10]. As the LSTM only has to be trained once the necessary number of training samples is the necessary number of numerical model evaluations and ideally much lower than the $4.32 \cdot 10^{7}$ model evaluations of the previous approach. A lower number of model evaluations would enable a more complex model.

Often, approaches that use synthetic data for training create datasets that combine real and synthetic data [59]. We avoided this to ensure a fair comparison with the previous optimization approach, which did not rely on any prior knowledge of real data either. Further, especially labeled human *in vivo* data are very scarce, so if the approach can provide reasonable performance using only synthetic data, this is preferable to demonstrate future clinical applicability. However, if enough data were available adding it to enhance the training dataset is a plausible option to further increase performance.

**FIGURE 2.**
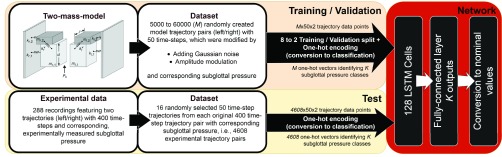
Schematic overview of the presented approach, data processing and architecture - Dropout is not displayed in the depiction. (Original image of the 2MM by Schwarz *et al.* [14]).

We used a LSTM network [2] and the Adam optimization algorithm [60] with gradient clipping [61] to minimize the cross-entropy loss over the class probability distribution produced by a softmax activation function. The neural network featured one LSTM layer with 128 cells followed by a fully-connected layer with $K$ outputs, where $K$ is the number of classification classes. Dropout was applied to the output of the LSTM layer with a probability of 0.5. A detailed overview of the architecture and the later presented data processing steps are given in Figure 2.

The approach is interesting both from the perspective of voice research as well as deep learning. In voice research the information of the subglottal pressure as well as other parameters can aid both research and possible future applications in a clinical setting. Unfortunately, with the current optimization procedure only simple numerical models can be employed in such an inverse problem and improvements in accuracy are impeded by the lower physical fidelity of these models. To illustrate, we employ an in-house developed software tool with highly optimized and parallelized implementations of the models, where 100 ms simulation time of the 2MM require about 1 ms of computation time on an Intel^®^ Core™i5-4590 processor. Even if a model requires just seconds of computation per evaluation, then performing more than 100 000 model evaluations for each new recording is not feasible, especially, in a potential clinical context, where timely results are indispensable. A deep learning approach, where a LSTM can be trained just once could render utilization of more sophisticated models possible.

From the deep learning perspective this approach is interesting because inverse problems in physical settings are not a frequent application domain for deep learning approaches. However, these inverse problems often rely on a large number of evaluations of the so-called forward model, typically a numerical model. Besides other problems, such as uniqueness of the solution and applicability of the model, the evaluations of the forward model are usually the computational bottleneck in these problems [38]. Deep learning can ease the computational burden, as it avoids a critical shortcoming of the optimization approaches: In classical optimization approaches the model evaluations used to solve one problem case - i.e. the optimization for one recording in our case - are not used after that case was solved and we start over. Replacing the complex numerical models in these inverse problems with a tailored neural network that directly outputs the results to the inverse problem can thereby significantly increase performance and might indicate that neural networks are suited to describe the underlying features of an inverse problem in a physical setting.

### E. Transfer Learning Approach

Training a neural network with data from a different source than the one that it is tested on is a well-established procedure. In particular, in computer vision problems pre-trained networks are regularly employed to avoid having to train the model and because the popular convolutional neural network’s performance translates well between different data sets [35], [36]. In our work we are interested in transferring the performance on a synthetic dataset to an experimental one, as only a small number of experimental samples is available. This is a harder problem than transferring to a different real data set, as synthetic data initially lack many features of the experimental data such as noise or segmentation and discretization errors. This section explores the tools we employed to close the gap between the datasets and to formulate the problem in an optimal way.

#### 1) Problem Formulation

The input of our problem can be considered a $2\times N$ matrix, where $N$ is the number of time-steps per trajectory. While the desired output $y$ is a scalar value and our problem thereby a regression, we convert it to a classification problem. This is similar to the procedures described by Oord *et al.* [62] and Zhang *et al.* [39]. Thereby, the class $C_{i}$ is

\begin{equation*} C_{i} = \{\,y\, | L_{i} \leq y < U_{i} \},\tag{4}\end{equation*}

where $L_{i}$ and $U_{i}$ are the class bounds. They are chosen in such a way that the classes were balanced, i.e. samples of each class in the training set occur with approximately equal frequency. As the used synthetic and experimental data were approximately normally distributed $L_{i}$ and $U_{i}$ are not equally spaced. Instead, the cumulative distribution function of the Gaussian distribution with mean and standard deviation of the training set is used to create balanced classes. $L_{0}$ was set to 392 Pa and $U_{n}$ to 1960 Pa, where $C_{n}$ is the class with the largest subglottal pressure values. Different class granularities were tested, in particular, classifying the data into between 138 and 1999 classes. The classification is later converted back to a scalar such that the estimation $\hat {y} = \frac {L_{i} + U_{i}}{2}$ for a classification result of class $C_{i}$.

#### 2) Data Preprocessing

The transfer between the synthetic data used for training and the experimental data can be eased in multiple ways. One approach is to try to process the experimental data to remove noise and reduce the volatility of the signal. However, this induces an information loss in the experimental data. Alternatively, the synthetic training data can be processed to be and look more like experimental data.

##### a: Subglottal Pressure Distribution

**FIGURE 3.**
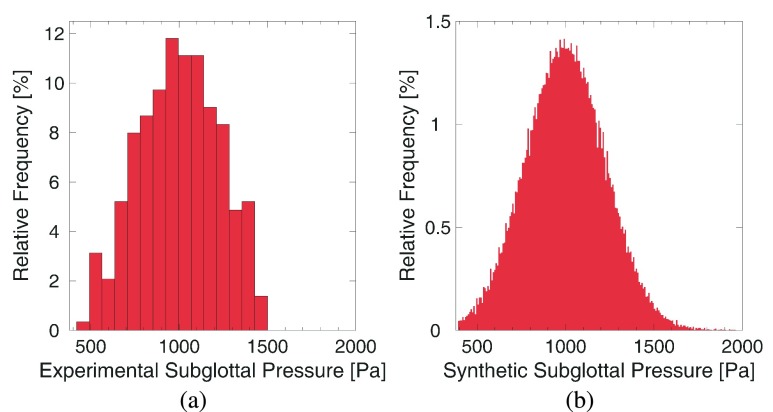
Distribution of subglottal pressure values in (a) the experimental data and (b) the synthetic data: Ideally, the synthetic and experimental data should be from the same distribution.

Randomly generated parameter configurations $q$ are used to create model configurations featuring random mass, stiffness, collision strength, and subglottal pressure. The synthetic data were generated using uniformly distributed values $q \in Q$, where $q = (Q^{m}_{l}, Q^{m}_{r},Q^{k}_{l},Q^{k}_{r},Q^{P_{s}},Q^{k^{c}})$ with $Q = [{0.3,4.0}]^{4} \times [{0.5,2.5}] \times [-1.0,8.0]$. To allow a fair comparison of the performance the parameter ranges of the training samples were similar to the ranges used for the optimization in the previous study [10]. One exception is the smaller range of stiffness values. This was necessary as configurations featuring high stiffness and high subglottal pressure can lead to numerical instability, which is acceptable in the optimization but not in the training data. Overall, it is preferable to have a parameter space as large as feasible to not bias the neural network towards certain parameter values or ranges. However, the parameter space is limited by the numerical stability of the model. A uniform distribution of mass, stiffness, and collision strength was assumed to ensure a rich enough dataset and because no population data such as mean and standard deviation of these parameters were available. The exception is the parameter $Q^{P_{s}}$, which affects the subglottal pressure and was sampled from a Gaussian distribution to match the experimental data as displayed in Figure 3. To create a realistic training data set $Q^{P_{s}}$ was sampled from a Gaussian distribution matching the mean and standard deviation of the experimental data. Subglottal pressure values and onset pressures are typically available for both human and animal population data [63]–[66]. Furthermore, it ensures that the classes $C_{i}$ are present with approximately equal frequency in the training, validation, and test set.

Trajectories not featuring the periodic trajectories, which are characteristic of sustained phonation, were discarded. The subglottal pressure values in the training data set were calibrated to account for the model’s tendency to underestimate [10].

##### b: Recording Length and Sampling Rate

The fundamental frequencies of the oscillations in the experimental dataset were between 70 Hz and 281 Hz. At 4000 Hz sampling rate an oscillation cycle was therefore approximately 14 to 57 data points long. Given the periodicity of the trajectories it is conceivable that less than 400 samples can be analyzed without a significant information loss. Furthermore, the present frequencies are well below the Nyquist frequency of 2000 Hz and downsampling instead of reducing the length of the trajectories might be an option as well but would require additional fine-tuning to avoid distorting the signal [67]. Hence, trajectories cropped to $N=50$ samples were used as input for the LSTM.

##### c: Artificial Noise and Amplitude Modulation

Another significant difference between the synthetic trajectories of the 2MM and the experimental trajectories is that the latter are affected by noise and that oscillations of real vocal folds feature some degree of amplitude modulation over time. The 2MM trajectories are often perfectly cyclic and noise-free. The experimental trajectories usually feature some measurement noise from either the recording or the segmentation. Furthermore, the complex vibratory patterns of real vocal folds feature slight amplitude modulation and aperiodic differences between oscillation cycles, such as slightly different fundamental frequency or vocal fold closing or opening speed.

**FIGURE 4.**
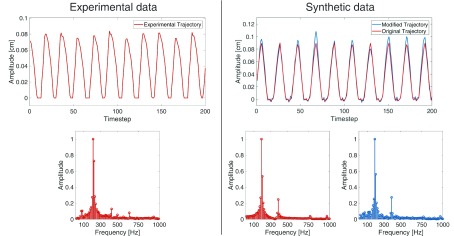
Exemplary trajectories to highlight the necessity and utility of the added noise and modulation - On the left an experimental trajectory and corresponding frequency spectrum are shown, on the right unmodified and modified synthetic ones. The modifications aim to ease the transfer between synthetic and experimental data.

We emulated the noise in the experimental data by adding noise from a Gaussian $\mathcal {N}(0,0.075\sigma)$, where $\sigma $ is the standard deviation of the trajectory that the noise is added to. Similarly, to replicate the amplitude modulation an envelope function was generated by a quadratic interpolation based on six points sampled from a Gaussian $\mathcal {N}(1,5\sigma)$. Each data point in a trajectory can then be multiplied with the evaluation of the interpolant to generate an artificially modulated signal. The purpose of this modulation was not to account for deficiencies of the model. Instead, the purpose was to add variability to the synthetic data to ease the transfer to real experimental data. Exemplary trajectories and the resulting modifications are displayed in Figure 4.

## IV. Results

This section presents the setup and the results obtained using the transfer learning approach described in the previous section.

### A. Setup

All results of this work were attained using the GPU version of TensorFlow 1.4 [68] on a GeForce GTX 1070 graphics card. The runtime of the 2MM was measured using an optimized and parallelized implementation written in *C#* on an Intel^®^ Core™i5-4590 processor.

Synthetic datasets of different sizes were tested. Each was split into a training and validation set with a ratio of eight to two. The test data were taken from the 288 experimental recordings. Only 50 data points of the trajectories were input to the network, but each recording featured 400 data points. Therefore, the test data set was artificially increased by picking 16 trajectories with 50 data points from each experimental recording by choosing a random starting point in the 400 data points. This led to a test data set containing 4608 samples. Note, that when we refer to the validation error in the following, it is the error computed evaluating synthetic data, and when we refer to the test error it is the error computed using experimental data. The MAPE is computed between the estimation of the subglottal pressure and the label of the validation sample or the experimental recording, respectively. The learning rate for the Adam optimization algorithm was set to 0.001, the batch size to 100. All results are averaged over ten different training runs to account for variability due to random initialization. Standard deviations refer to the ten runs. All hyperparameters were determined by hyperparameter search. After initial convergence, training was stopped if the validation loss did not decrease for five epochs to avoid overfitting or after a maximum number of 400 training epochs.

### B. Experimental Results

This section explores the impact of the size of the used synthetic data set and the classification granularity.

#### 1) Dataset Size

One factor of utmost importance in our approach is the necessary amount of training data. One of the reasons this approach is interesting is to avoid the high number of numerical model evaluations necessary in previous approaches [10], [55]. We therefore investigated the accuracy and loss results for training with datasets containing between 5000 and 60 000 trajectory pairs and labels.

Table 1 displays the results on the validation and test data after training a network with different dataset sizes. The discrepancy between the training and validation loss for the networks trained with fewer samples is a clear indicator that overfitting was a problem with smaller datasets. With 40 000 samples the overfitting problems are less severe and the MAPE on the test set is best. Using 60 000 samples, however, a better validation accuracy and even less overfitting are observed.

**TABLE 1:** Results of Training on Datasets of Different Size: the Classification Used 1007 Classes. The Losses Indicate Overfitting is a Problem With Less Than 40 000 Samples

	Training		Validation		Test
#Dataset samples	#Training epochs	Loss	Loss	MAPE [%]	MAPE [%]
5 000	287.0	1.23	10.63	18.59	25.29
10 000	147.0	4.21	7.97	16.01	24.71
20 000	78.0	5.05	6.61	9.43	21.49
40 000	53.4	5.57	6.08	8.07	21.21
60 000	44.8	5.62	5.98	7.52	21.66

#### 2) Classification Granularity

Another important parameter is the classification granularity, which was introduced in Section IV.A. In practice, we tested values leading to between 138 and 1999 classes. Note that a higher number of classes leads to higher possible accuracy when converting back from the classification to the subglottal pressure values corresponding to each class. However, more classes also result in fewer samples for each class in the training set and a harder classification problem.

Table 2 contains the results for different classification granularities. Overall, 1007 classes featured the best results on the test set with a MAPE of 21.66%, but required longer training and featured slightly worse validation accuracy than the simpler classification with less classes. For 1999 classes the results did not improve anymore but the number of training epochs significantly increased. Note that higher losses are expected with more classes and are not necessarily indicative of less accuracy as each class represents a smaller interval of subglottal pressure values for a higher number of classes. All following results refer to a classification granularity leading to 1007 classes with 40 000 samples.

**TABLE 2:** Results of Different Classification Granularities Using a Dataset of 60 000 Samples: Between 138 and 1999 Classes Were Tested. The Irregular Numbers of Classes Occur Due to the Class Choices, Which Ensure Approximately Equal Class Representation. Losses are not Comparable Between Rows Due to the Different Numbers of Classes

	Training		Validation		Test
#Classes	#Training epochs	Loss	Loss	MAPE [%]	MAPE [%]
138	38.1	3.58	3.73	7.02	23.95
212	39.1	4.27	4.55	7.10	23.43
410	41.5	4.86	5.23	7.33	23.45
1 007	44.8	5.62	5.98	7.52	21.66
1 999	57.7	6.38	6.83	8.03	21.65

### C. Subglottal Pressure Estimation

**FIGURE 5.**
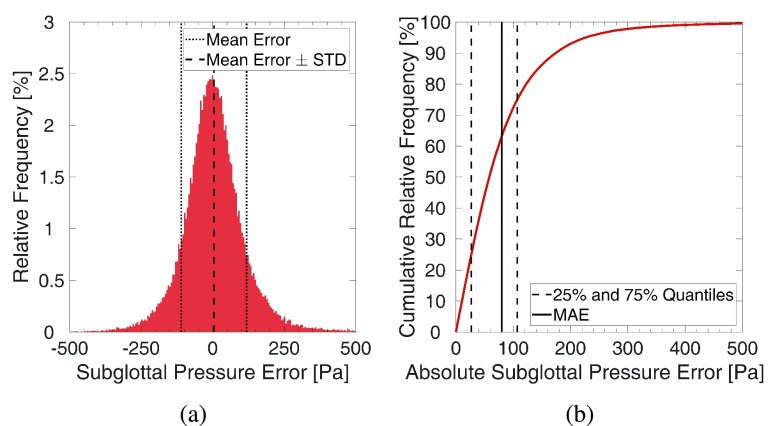
Relative and cumulative distribution of subglottal pressure errors on the validation set using a dataset of 40 000 samples and classification into 1007 classes.

Overall, training with 1007 classes and a dataset of 40 000 samples led to a MAPE of the subglottal pressure on the validation set of 8.07% (SD = 0.25%). The previous study [10] reported a MAPE of 4.9% on synthetic test data. The corresponding MAE was 80.3 (SD = 2.65) Pa. Figure 5 displays the relative and cumulative distribution of the subglottal pressure errors on the validation set. Errors clearly follow a Gaussian distribution. 25%, 50%, and 75% of errors were smaller than 26.8, 58.2, and 107 Pa respectively. Note that the synthetic data in the previous study were not affected by noise and the amplitude modulation employed in this work.

**FIGURE 6.**
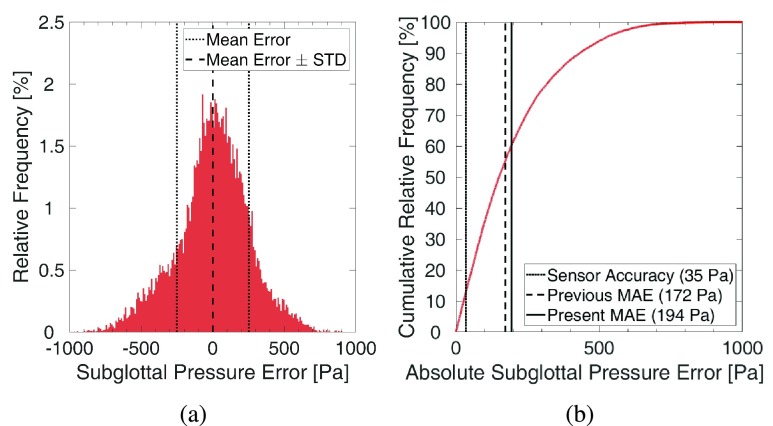
Relative and cumulative distribution of subglottal pressure errors on the test set using a dataset of 40 000 samples and classification into 1007 classes: Vertical lines in (b) indicate reproduction accuracy of the used pressure sensor, and mean errors of the previous approach and our approach.

On the test set, i.e. the experimental data, the achieved MAPE was 21.21% (SD = 0.89%) with an MAE of 194 (SD = 7.35) Pa. The previous study [10] achieved 17.65% MAPE and 172 Pa MAE. The mean experimental subglottal pressure was 996 Pa. Figure 6 displays the relative and cumulative distribution of the subglottal pressure errors on the test set. The distribution of the error values is slightly skewed with a mean of 1.41 Pa and standard deviation of 252 Pa. The distribution resembles a Gaussian distribution. The vertical lines in Figure 6b indicate the reproduction accuracy of the pressure sensor in the experiment and MAE of the previous approach [10] and the new approach. 12.9% of errors were smaller than the sensor’s reproduction accuracy. Note that 55.2% and 60.3% of errors were smaller than the mean error values of the previous and new approach, respectively. The distribution of absolute errors is positively skewed indicating that particularly unsuccessful predictions are impacting the mean errors proportionally more. 25%, 50%, and 75% of errors were smaller than 69.0, 150.4, and 276.4 Pa respectively.

## V. Discussion

Overall, our results indicate strongly that the presented approach is well suited to ease the computational burden arising from the need to evaluate the forward model many times to solve this inverse problem. Table 3 displays a comparison of the presented approach with the approach from the previous study [10].

**TABLE 3:** Comparison of the Previous Approach [10] and Our New Approach: Values Marked With an Asterisk Were Extrapolated

	Original approach	LSTM approach	Speedup / Increase
2MM evaluations	43 200 000	40 000	$\times1\,\,080$
2MM runtime [s]	8 415*	6.20	$\times1\,\,357$
Training time [s]	-	569	-
Evaluation time per sample [s]	5.85	$1.03 \cdot 10^{-4}$	$\times56\,\,796$
MAPE [%]	17.65	21.21	+ 3.56
MAE [Pa]	172	194	+ 22

### A. Runtime

In terms of total runtime and necessary model evaluations the new approach outperforms the previous work. We require 1080 times less model evaluations. The evaluation time per sample decreased by a factor of 56 796. It is critical to note that the much lower number of model evaluations in this approach would allow for a computationally more demanding numerical model than previous approaches. A model taking seconds to compute was not feasible with previous approaches but would be with the presented one, especially since the time to train the network would not increase. With a model requiring 2 seconds per evaluation, e.g., the generation of the training data would take less than a day while the evaluation and training time of the LSTM should be comparable.

### B. Parameter Estimation and Inverse Problems

The error estimating the subglottal pressure for the synthetic and experimental data is only slightly worse utilizing a neural network compared to the previous approach [10]. On average, the MAE was 22 Pa higher than with the previous approach (172 vs. 194 Pa). For reference, the reproduction accuracy of the pressure sensor used in the experimental setup (Kulite XCS-093) is approximately 35 Pa. The mean experimental subglottal pressure was 996 Pa. Hence, the increase in the MAE is comparable to an inaccuracy of the measurement equipment, but relatively insignificant compared to the measured subglottal pressure.

Note that the accuracy of the presented approach estimating the subglottal pressure for experimental data is limited by the accuracy of the numerical model, i.e. the 2MM, used for the training. It is conceivable that employing a more sophisticated numerical model can significantly increase the accuracy of the neural network, if it is trained on synthetic data from such a model. The errors estimating the subglottal pressure display low skewness, which indicates that factors that could introduce a bias such as the posterior gap present in the recordings [50], [51] were not a problem for the LSTM. Furthermore, given the adaptability of this approach, an application to estimate other parameters is conceivable. Overall, it is able to estimate the subglottal pressure with a comparable precision as the previous approach at a significantly reduced computational cost.

By training on synthetic data from a numerical forward model, we are thereby able to significantly decrease the number of forward model evaluations. The basic idea of this is likely transferable to other inverse problems, where a high number of numerical model evaluations is required.

### C. Benefit of the Approach

The main focus of this approach was to alleviate the computational burden of a parameter estimation using a numerical model without sacrificing the demonstrated accuracy of the model [10]. The main reason for the high computational demand are the high number of model evaluations during the optimization and the necessity to start from scratch for each new recording. The presented approach circumvents these problems by employing a neural network for the estimation. Overall, the following benefits were found in direct comparison with an optimization approach:

• Vastly decreased computation time for the subglottal pressure estimation compared to optimization approach
• Accuracy comparable to an optimization of the parameters of a numerical model
• Required just 40 000 model evaluations
• Training with a sophisticated model, e.g., a 2-D Navier Stokes model likely feasible
• No model evaluations required after one-time training.

## VI. Conclusions and Outlook

The presented approach allows the prediction of vocal fold parameters utilizing a neural network to provide estimation with a comparable accuracy as previous approaches at a fraction of the computational cost. Knowledge of the subglottal pressure will help in diagnosis as it is indicative of disorders [4], [5]. Quantification of such parameters can also help in treatment selection [14]. The approach can enable the use of more complex models, which can aid in increasing accuracy and is instrumental in bridging the gap from models, which require a supercomputer to run to ones, which are applicable in daily clinical routine. In the future, as high-fidelity models [30], [32] become computationally less demanding, this approach can aid their clinical application.

A similar approach to estimate other parameters such as vocal fold stiffness is conceivable, which will be a topic of future research. Future work will also investigate, how much a model with a more sophisticated geometry and airflow model such as a 2D Lattice Boltzmann or Navier-Stokes approach can increase accuracy.

More generally, inverse problems such as the presented one are often ill-posed and have a high computational burden if adequate forward models are to be employed [38]. The presented approach demonstrates that the computational burden can be eased significantly by training a neural network, where the forward model is only used to train a neural network. Thereby, the training requires no hand-labeling, relies solely on synthetic data and no further forward model evaluations are necessary. It also highlights the potential of applying deep learning techniques to deal with computationally complex simulations. It would be interesting to investigate comparable inverse problems and find out if and how much they may profit from a similar approach.
